# A diet rich in polyunsaturated fatty acids as supportive therapy in the treatment and prevention of psychotic disorders

**DOI:** 10.1192/j.eurpsy.2023.2306

**Published:** 2023-07-19

**Authors:** R. Sitarz, D. Juchnowicz, K. Karakuła, A. Forma, J. Baj, J. Rog, R. Karpiński, A. Machrowska, H. Karakuła-Juchnowicz

**Affiliations:** 1 Chair and I Department of Psychiatry, Psychotherapy and Early Intervention, Medical University of Lublin, 20-059 Lublin, Poland, Chair and I Department of Psychiatry, Psychotherapy and Early Intervention, Medical University of Lublin, 20-059 Lublin, Poland; 2 Department of Psychiatry and Psychiatric Nursing, Medical University of Lublin, 20-059 Lublin, Poland, Department of Psychiatry and Psychiatric Nursing, Medical University of Lublin, 20-059 Lublin, Poland; 3 Department of Forensic Medicine, Medical University of Lublin, 20-059 Lublin, Poland, Department of Forensic Medicine, Medical University of Lublin, 20-059 Lublin, Poland; 4 Department of Anatomy, Medical University of Lublin, 20-059 Lublin, Poland, Department of Anatomy, Medical University of Lublin, 20-059 Lublin, Poland; 5 Department of Dietetics, Institute of Human Nutrition Sciences, Warsaw University of Life Sciences, 02-776 Warsaw, Poland, Department of Dietetics, Institute of Human Nutrition Sciences, Warsaw University of Life Sciences, 02-776 Warsaw, Poland; 6 Department of Machine Design and Mechatronics, Faculty of Mechanical Engineering, Lublin University of Technology, Poland, Department of Machine Design and Mechatronics, Faculty of Mechanical Engineering, Lublin University of Technology, Lublin, Poland

## Abstract

**Introduction:**

Proper nutrition with fats has a protective effect on the functioning of the nervous system. However, a disturbed ratio of essential polyunsaturated fatty acids supply is nowadays a common phenomenon. A diet overloaded with saturated fats and a shortage of those essential ones in the company with possibly some unfavorable genetic endowment could lead to the release of psychosis from the framework of diet defined by nature for thousands of years.

**Objectives:**

The study aims to review the literature to assess the influence of supplementation with polyunsaturated fatty acids in the occurrence of psychotic disorders prevention, as well as their impact on remission prolongation.

**Methods:**

Literature review in PubMed, Google Scholar, and Web of Science using the keywords [psychosis] OR [psychotic] OR [schizophrenia] OR [unipolar] OR [bipolar] OR [schizoaffective] OR [depression] OR [manic] OR [hypomanic] OR [mania] OR [hypomania] OR [first episode psychosis] OR [ultra-high risk] OR [UHR] AND [polyunsaturated fatty acids] OR [PUFA] OR [prostaglandin] OR [phospholipid] OR [phospholipase A2] OR [arachidonic acid] OR [linoleic acid] OR [alpha-linolenic acid] OR [omega-3] OR [omega-6] OR [nutrition] OR [diet]. The review included original articles, reviews, systematic reviews, meta-analyses, and case reports from 1977-2022 in Polish and English.

**Results:**

86 articles devoted to diet and nutrition in psychotic disorders were analyzed. Patients with schizophrenia, bipolar disorder, and schizoaffective disorders exhibit deficiencies in polyunsaturated fatty acids. Such results may indicate compliance with David Horrobin’s theory of the psychotic disorders development in predisposed individuals.

**Conclusions:**

Supplementation with polyunsaturated fatty acids may be a chance for a selected group of patients to prolong remission but also hope to prevent the occurrence of psychotic disorders in particularly vulnerable individuals.

**Disclosure of Interest:**

None Declared

